# “When You Are Holding a Pen You Think Deeper about What You Write.” Comparing the Experiences of Completing Paper- and Computer-based Participant-aided Sociograms

**DOI:** 10.1177/1525822X261434661

**Published:** 2026-04-05

**Authors:** Dorottya Hoór, Vuyiswa Nxumalo, Bernie Hogan, Guy Harling

**Affiliations:** 1 Dorottya Hoór, University College London, Mortimer Market Centre, London, UK; 2 AHRI, Durban, South Africa; 3 University of Oxford, Oxford, England, UK

**Keywords:** personal networks, network data collection, participant-aided sociograms, Network Canvas, journey-based analysis

## Abstract

Past personal network research has highlighted the many trade-offs in data collection strategies but has largely overlooked respondents’ perspectives on the interview experience. We use a within-subjects research design to compare respondents’ experiences with pen-and-paper sociograms and a comparable computer-assisted approach using Network Canvas, among young people in rural KwaZulu-Natal, South Africa. Data were collected through five focus groups (30 participants in total) and analyzed using thematic analysis and a novel journey-based approach. Our results highlight key experiential trade-offs between the analog and digital methods focusing on cognitive burden, emotional investment, and practical constraints.

## Introduction

Social networks are at base constructed from two sets of objects: nodes and edges, typically people and the relationships between them. While some networks (such as a classroom) are bounded, allowing data collection from all members, we often sample from a large set of potential nodes and then measure the immediate relational neighborhood of sampled respondents (or egos). Within this latter approach—often called personal network analysis or egocentric network analysis—researchers are often interested in respondents’ perceptions of relations between their nominated peers (or alters) since how these alters are connected to one another makes a qualitative difference to egos’ sense of their social world (Fischer 1982; Wellman and Wortley 1990).

Researchers have explored a variety of approaches to collecting ties between alters, appreciating that such questions can be both time consuming and can also pose a significant cognitive burden to respondents (Burt 1984; Herz and Petermann 2017; Marsden 2011). In recent decades, researchers have sought to reduce this burden through different visual network data collection tools, rather than applying a dyad census. These methods have shortened the overall process, provide a cognitive aid for depicting relationships, help maintain an overview of relationships and thus aid alter recall, and also better hold respondents’ attention (Bellotti 2016; Hogan et al. 2007; Hollstein et al. 2020; McCarty and Govindaramanujam 2005).

Early visual data collection methods converged on a concentric circles design for arranging social ties, radiating out from the center (Antonucci and Akiyama 1987; Spencer and Pahl 2007). Analog and digital approaches to network data collection have since coevolved using this structure. For example, McCarty’s digital EgoWeb, developed in the early 2000s (McCarty and Govindaramanujam 2005), was a key reference point for the subsequent pen-and-paper participant-aided sociogram (Hogan et al. 2007). Further refinements have emerged including use of “magnetic poetry” and dry erase markers (Kuhns et al. 2015), Lego bricks (Stys et al. 2022), and open-ended network drawings (Marsden and Hollstein 2023). In parallel, digital approaches have grown by taking advantage of efficient data processing, cost-effective touch screen displays, and web standards for cross platform technologies (Hogan et al. 2016; Marsden and Hollstein 2023). Notable digital software includes EgoNet (Borgatti 2006), GENSI (Stark and Krosnick 2017), Network Canvas (Hogan et al. 2016) and VennMaker (Gamper et al. 2012). These digital methods appear to simplify network data collection, improve data quality, and ease both respondent and researcher burden (Hogan et al. 2016).

While research comparing digital and analog approaches within the same study is limited, evidence is emerging of trade-offs between paper-based and digital methods. On the one hand, digital tools (specifically Network Canvas) appear to produce high-quality data, with comparable reporting of alter numbers and sensitive information (e.g., sexual partners, shared drug use), while taking substantially less time to complete (Birkett et al. 2021; Hogan et al. 2016, 2020).

Digital tools also appear to generate reliable longitudinal data, reflected in no reduction in the number of reported contacts over waves—suggesting they may reduce motivated underreporting (Birkett et al. 2021; Hogan et al. 2020). Additionally, participants also report these methods to be easy to understand and use and report high user satisfaction (Eddens and Fagan 2018; Hogan et al. 2016; Tubaro et al. 2014).

On the other hand, digital tools may lead to fewer alter–alter ties being reported, particularly when relationships between alter pairs are not asked about individually (i.e., no tie census), which impacts network structure measures (Eddens and Fagan 2018; McCarty and Govindaramanujam 2005; Tubaro et al. 2014). Furthermore, digital methods can be unsuitable when electronic data collection is not allowed and may also incur significant up-front costs (Hogan et al. 2007; Schiffer and Hauck 2010).

While practical trade-offs between paper-based and digital tools have been identified, less attention has been given to differences in interview experience from the respondent’s perspective. These experiential qualities may impact data quality, subject retention and researchers’ ethical commitments (Hogan 2021), especially for sensitive topics. When respondents’ experiences are considered, the focus is often on them as technology users—measuring ease of use, efficiency, and enjoyment—but overlooking the subjective experience of reporting on their own personal networks in a research setting (Eddens and Fagan 2018; Hogan et al. 2016; Tubaro et al. 2014). We therefore aim to understand participants’ differential experiences completing analog and digital personal network data collection methods.

We do this by using a comparative within-subjects research design to assess respondents’ experiences when completing a pen-and-paper sociogram and a comparable computer-assisted screen-based approach using Network Canvas within the same research protocol in the resource-constrained setting of rural KwaZulu-Natal, South Africa. We draw on qualitative data from 30 young adults from a population where smart phone use is ubiquitous, in five interviewer-facilitated focus group sessions, and apply journey-based analytical methods to explore respondents’ positive and negative experiences of using both methods, focusing not on variations in data quality directly, but on the experience of providing information using these two methods.

## Methods

### Data Collection

Our data arise from preparatory work for Sixhumene (“we are connected” in isiZulu), a longitudinal study examining social support networks and sexual health of young people conducted at the Africa Health Research institute (AHRI) in rural KwaZulu-Natal, South Africa (Nxumalo et al. 2022). Given limited digital uptake locally, the project aimed to test the acceptability and feasibility of digital versus analog personal network data collection tools. We collected data through five focus group sessions in Mtubatuba, South Africa, with 30 participants (17 female, 13 male) aged 18–30, conducted in 2019 and 2022 (following Covid-related delays). All interviews were conducted in isiZulu by experienced researchers. Four out of five focus groups were gender stratified and sampled from two communities, with some of them tangentially knowing each other; the fifth was composed of community-based peer navigators who work together to link young people to health interventions in the community.

We implemented a within-subject design to compare digital and analog experiences. All participants completed the same network generation exercise twice on the same day, in analog using a paper participant-aided sociogram (A3 size), and digitally via Network Canvas on tablet computers. Both sessions were led by facilitators, where, after an initial introduction, participants completed the paper version step-by-step following verbal instructions to the whole group and the tablet version by following on-screen instructions. For both versions, facilitators were available to resolve concerns throughout. In each focus group, participants were randomly assigned into one of two groups, with one group completing the digital method first and the other beginning with the paper approach.

After completing the first network exercise, facilitators led each group in a structured discussion using cognitive interviewing techniques to capture participants’ cognitive processes as they completed the exercise. Upon completing the second network exercise, the small group discussion process was repeated with a focus on differences in ease of use and preferences for each approach. All discussions were digitally recorded, transcribed, and translated from isiZulu to English for subsequent analysis.

The two data collection methods followed nearly identical network elicitation processes based on commonly used elements of personal network questionnaires: name generators (important person and seven exchange-based questions (see Appendix); placing alters on concentric circles; name interpreters; and edge interpreters (Table 1). The only differences between the two methods were respondents having to indicate alter–alter ties earlier in the tablet-based version due to software constraints within Network Canvas. Network Canvas networks were recorded as a screen shot and exported to a secure server, while paper-based sociograms were scanned and securely stored.

**Table 1.** Overview of paper-based and digital network elicitation workflow during the focus group and their corresponding journey stages.

Network elicitation process	Data goal	Paper implementation	Digital implementation	Journey Stages
Name generator: generating/obtaining network members	Listing important people, starting with most important	Write alters for each question in different colors	Enter alters into Network Canvas interface	Stage 1
	Exchange-based name generator questions: emotional, practical, financial and socialization support	Write alters for each question in different colors	Enter alters into Network Canvas interface	
	Nominating alters they might communicate with only rarely but make a big difference in their lives, and feel close to but have not been named so far	Write alters for each question in different colors	Enter alters into Network Canvas interface	
Name interpreter	Obtaining (emotional) closeness	Write alters on concentric circles (target)	Place alters on concentric circles (target)	Stage 2
Alter-alter ties	Obtaining alter-alter relations	-	Connect alters who speak at least once a week by tapping both names in turn	Stage 3
Name interpreter	Obtaining alter attributes on age, gender, household membership	Write with different colors in the vicinity of alters names	Tap on alters living in the same household; enter age as a number; tap to select gender	Stage 4-6
Edge interpreter	Frequency of seeing alters by selecting from a pre-determined set of six options, ranging from less than once a month to almost every day	Write 2 or 3 letter codes near alters	Sorting names into bins corresponding to each option	Stage 7
Alter-alter ties	Frequency of alter-alter interaction, using the same categories pertaining to the frequency of their communication with one another	Connect alters with different types of lines	Tie survey, including selection of one of six frequency options for alter pairs previously indicated to have a relationship	Stage 8
Name interpreter	Obtaining alter attributes on whether alter is a family member and whether they live in the same isigodi (geographical area)	Add 1 or 2 letter notation for family membership; circle names with different colors depending on where alters lived	Sort names into two groups (family member or not; lives in the same isigodi or not)	Stage 9-10

All participants provided written informed consent prior to participation. The study was approved by the University of KwaZulu-Natal’s Biomedical Research Ethics Committee and University College London’s Research Ethics Committee, as well as by AHRI’s Community Advisory Board.

### Analysis

After transcribing and translating discussions, we coded the text using Thematic Analysis (Braun and Clarke, 2021). We first used an inductive open coding process that generated codes relating to both positive and negative sentiments associated with participants’ experiences. We classified these experiences into three major themes pertaining to different aspects of the data collection process: cognitive burden/ease; emotional experiences; physical affordances/constraints.

We arranged these experiences using the Trajectories framework from the Human Computer Interaction (HCI) literature (Velt et al. 2017). This framework is designed for tasks where the end goal is set by the researcher, but the steps taken can vary by participant. Our tasks (Table 1) always follow the same sequence but vary in complexity depending on the size and density of the nominated network. Trajectory analysis aligns steps across participants to understand which steps in the journey elicit qualities of interest (such as comprehensibility or sentiment). We chose to align these journeys on the relatively general metric of sentiment (Medhat et al. 2014). Sentiment is commonly disaggregated into positive and negative aspects, allowing for ambivalence when someone both likes and dislikes elements of the same task. Usefully, sentiment strength can be understood from participant’s statements about each stage. Recognizing that sentiment lacks granularity, which can limit interpretability, we believe this coarse but clearly detectable measure is warranted, given our lack of prior beliefs about which stages and modalities would elicit which sentiments. We augment our interpretation with quotes from our thematic analysis.

For our trajectory analysis, we identified 10 discrete stages of the interview journey across the key components of personal network data collection process (name generators, name interpreters, etc.): (1) name generator; (2) emotional closeness; (3) whether alters know each other; (4) whether alters live in the same household; (5) alters’ gender; (6) alters’ age; (7) frequency of seeing alters; (8) frequency of alters talking to each other; (9) whether alter is a family member; and (10) whether alter lives in the same *isigodi* (local community) (Table 1). To capture participants’ entire interview experience, we included two additional stages in our analysis: (11) comparing the two data collection methods; and (12) participants’ reflection on their networks.

 We mapped sentiments onto a linear diagram of corresponding journey stages, creating two temporal heat maps of the personal network data collection process—one for each method. The resulting figure shows journey stages on the x-axis, with each participant’s positive sentiments (in blue) in a single row above the axis and their negative sentiments (in red) in a single row below the axis. Color saturation reflects more frequent positive or negative comments about that stage from the participant.

## Findings

### Key Dimensions of Positive and Negative Experiences

We identified three key thematic dimensions that captured participants’ negative and positive experiences throughout data collection under both modalities: (1) cognitive burden or ease; (2) emotional experiences; and (3) physical affordances or constraints.

#### Cognitive Burden or Ease

As in most data collection processes, the cognitive burden of personal network data collection varied across respondents and interview sections. The greatest burden most commonly arose when deciding on the boundaries of participants’ networks and when respondents lacked factual knowledge about alter or tie attributes. For some participants, boundary setting came naturally:

It was easy because these people are the people I look up to and rely on. So, when I am writing here, it just occurred to me that these are the people I must write here, they are the people who are important to me. [Male, age 19, paper-based method first, first discussion]

However, others struggled to translate their complex social realities, such as inconsistent support, into well-defined support networks:

The person which was difficult for me place on the map, is my uncle. I don’t know how I can explain it, but sometimes he supports us in that way, but it will be only for a week and then after that he disappears. [Female, age 21, paper first, first discussion]

Similarly, while some participants reported no problem providing alter attributes, others reported not having sufficient knowledge, such as alters’ age:

It’s not everyone who knows everyone’s age. So, you may ask me about someone else’s age and find that I don’t know their age. I don’t think it’s necessary to ask about their age, for instance I know my mother’s age but someone else doesn’t know their mother’s age. [Male, age 28, tablet-based method first, second discussion]

Particular difficulty arose when reporting how often alters communicated, especially when egos lived elsewhere:

The way they communicate is difficult [errr] the ones who are there are the ones who communicate there the ones who live together, yes, most of the time the ones who do not live there[..] it happens for days, one call after another [. . .] so it was difficult for him to say that they call a few times a week, coz for one week they just call every week and other week not a day goes by without them calling. [Male, age 20, paper first, first discussion]

#### Emotional Experiences

The personal network data collection process often evoked both positive and negative emotional sentiments in participants, most commonly early in the interview (when deciding on network boundaries and placing alters on the targets) and in the final reflective stage. For many participants, seeing their social world was a rewarding experience, which prompted a sense of gratitude toward people in their lives.

I realized that there are people in my life that I should not lose, there are people [. . .] are people in my life that I would not be here if it was not for the people I wrote here. [Male, age 19, paper first, first discussion]

However, in some cases, the name generator process made participants realize that their support networks may be inadequate and their social relations may lack reciprocal support.

What surprised me was that, sometimes you can say that you love people and they help you, and in the end, they will turn against you and they help you with nothing when you need them. [Female, age 24, paper first, first discussion]

The name generator process sometimes also highlighted unfulfilled support expectations, particularly among kin, which caused participants distress—especially when traditional support channels (e.g., maternal support) were disrupted.

Many of my relatives, my uncles, and my dad too, I was hurt that he is not close to me as he is the first one there, like my aunt, granny, but he is below, even my friends outshine him, I prefer them than him because the support is not enough, he call when it suits him, I do not like the way my uncles behave, they were supposed to be included in the map but I didn’t do that due to their bad painful actions, so they were supposed to be included because I no longer have a mother, my mother used to help them with everything but they fail to help me with anything, you see, that was it. [Female, age 23, paper first, first discussion]

#### Physical Affordances and Constraints

Finally, participants reported both positive and negative experiences regarding the physical affordances and constraints of both methods (Norman 2004). These grouped into four main themes: (1) ease and speed of data collection; (2) visual organization/clarity of network data; (3) flexibility of data collection; and (4) data security. First, participants noted that the digital approach was faster and less physically complex.

I found it easy as we used to do it on the phone, everything is fast and time is short. [Male, age 23, paper first, second discussion]

When we were handwriting, it took a long time, we kept changing pens while writing and this one [tablet] keeps clicking for a brief time. [Male, age 19, paper first, second discussion]

Second, the digital approach allowed easier placement of alters, name interpreter information and connecting of alters, while also keeping the sociogram legible—largely a function of the physical constraints of the paper method.

For me, I think it can be better if the map is broader because there are many things that we are supposed to. . ., there are many people that we are very close to, people that we love but we are unable to fill this map properly. [. . .] This thing that explains whether a person is family and so on, it was eventually squeezed, you see, it is better if the map could be wider, according to me. [Female, age 24, paper first, first discussion]

When drawing the lines, you have to check where it is going, so that you can avoid the mess. While with this one [tablet], you just do take it from here to there, you don’t need to get it neat. [Female, age 30, tablet first, second discussion]

Third, the digital method provided greater flexibility to change one’s mind during the process, and to correct data entries participants perceived as mistakes, without diminishing the visual clarity of their networks.

With the tab, if you make a mistake it is easy to delete quickly, so you cannot see that they made a mistake and the paper is already scratched, now it is dirty. [Male, age 19, paper first, second discussion]

Also you can let’s say you have skipped or missed something, you can make changes at that time, here how can you erase when it’s already a mess. [Female, age 30, tablet first, second discussion]

Finally, as participants perceived their networks to be sensitive information, they expressed concerns about its safety, particularly other people accessing the copy of their paper-based networks.

With the tab, it will save everything, but with the paper it will be ruptured, it can get lost and damaged and all that. Whereas with the tablet, I can save all the information. [Male, age 28, tablet first, second discussion]

And that with the tablet I can save and then submit and it also notifies me when I’m done with my interview. Whereas with this one, [paper] I can come across my enemy and they can take my paper away then it’s a mess [group laughter] then I have to start afresh. [Female, age 26, tablet first, second discussion]

### Journey-based Comparison of the Two Methods

**Figure 1.**
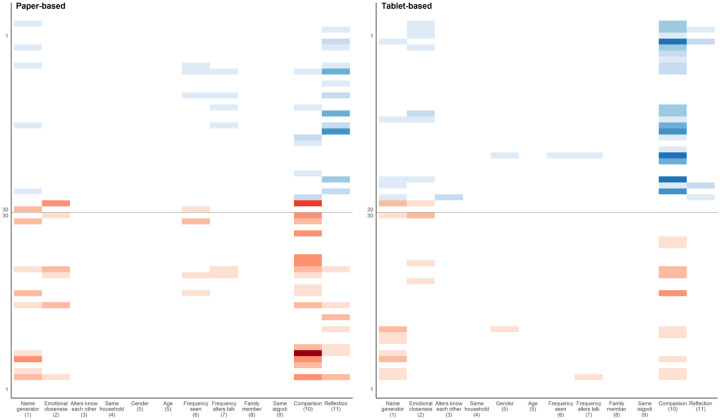
Heatmap of sentiments arising from the social network interview journey. The figure shows the stages of the network interview on the x-axis for both methods, with each participant’s experiences shown as two mirrored rows above and below the x-axis. Positive experiences are above the axis in blue; negative experiences are below the axis in red. More saturated hues reflect a larger number comments about that stage from that respondent.

Participants named similar numbers of alters across methods: a mean of 12.9 (range 6 to 24) using Network Canvas and 12.0 (range 6 to 26) using paper. Further similarities but also differences were apparent when comparing participants’ journeys across methods (Figure 1). Similarities included uniformly finding answering name generator questions and placing alters on the network map the most demanding processes, as shown by a concentration of negative sentiments. These negative sentiments are predominantly underlined by cognitive burden and negative emotional experiences. Participants found it difficult to define the boundaries of their personal networks, and thus to select who to include in their networks, especially for ambivalent relations. Similarly, deciding on ambivalent alters’ closeness on maps also generated cognitive and emotional burden.

Clear differences emerged at the end of the journey. Most participants overall preferred Network Canvas, reflected in concentrations of positive sentiments for the tablet-based method and negative sentiments for the paper-based one. Their preference mostly reflected the ease and speed of the digital process, often underpinned by its physical affordances as described above. The only digital drawback mentioned was size constraint, imposed by smaller screen compared to the A3 paper used for the analog method. Nevertheless, some participants still preferred the paper-based method, largely due to the depth of thought the method induced:

The paper one took longer because you had to write everything. [. . .] I prefer [that] because when you are holding a pen you think deeper about what you will write and keeps you involved with what you are doing. [Male, age 18, paper first, second discussion]

This positive view of the paper method was reinforced in the last stage of the journey (participants’ reflection on their networks): While both methods prompted participants to reflect emotionally on their networks, this experience was much more common with the paper-based method. The paper-based process was also more likely to induce mixed emotions, including inadequate support receipt and difficult relationships. This aligns with the reasoning behind many participants’ preference for the tablet-based method, explained by one of them as “[. . .] you do not need to think too much, you type, you enter.” This heightened but ambivalent engagement with the paper-based method was also seen mid-journey (provision of alter and tie characteristics), with more comments about both cognitive ease and difficulty; the section went largely uncommented on in the tablet-based version.

## Discussion

In this qualitative study of how participants’ differentially experience paper-based and digital methods of reporting on their personal social networks, we combined thematic analysis with a journey-based visualization approach to identify key insights relevant to future network data collection projects.

First, we were able to clearly categorize respondents’ experiences during the process using three overarching themes. One theme (physical affordances and constraints) reflects the physical process of engaging with the study tools. The other two themes capture experiences relating to respondents’ mental or cognitive processes—the challenges of completing the research task (cognitive burden or ease) and immediate impressions of seeing self-representational data (emotional experiences). Respondents’ consistent and sometimes unsolicited remarks concerning the emotional impact of constructing and perceiving their networks, and the frustration of not being able to complete tasks to their own satisfaction (e.g., establishing a reliable boundary for who is in the network), highlight the importance of respondents’ subjective experience in completing personal network interviews—both for data quality and researchers’ ethical care obligations to the participants. An interview experience will be inappropriate either if the data generated are poor quality, or if the respondent suffers unnecessary harm in the process. It is thus vital that those designing structured interviews move beyond a narrow focus on participants’ experiences as technology users, to incorporate potential emotional impacts of the process (D’Angelo and Ryan 2021; Eddens and Fagan 2018; Hogan et al. 2016; Tubaro et al. 2014).

Second, our journey-based analysis reinforced that the step of identifying personal network members and organizing them in relation to one another, even when using visual tools, generates substantial cognitive burden on respondents (Burt 1984; Marsden 2011; Marsden and Hollstein 2023). This highlights the importance of methods to reduce cognitive burden such as careful selection and wording of name generator questions and employing narrative interview and other creative techniques to capture participants’ networks. Cognitive burden can be a particular problem for measuring alter–alter interactions. Our analysis reinforces the fact that visual methods reduce this burden while yielding very similar sizes of networks (Hogan et al. 2016).

Third, our journey-based comparison revealed a key trade-off between methods from respondents’ point of view. On the one hand, the digital method was preferred for its ease, speed, flexibility, and clear network display. On the other hand, the paper-based version prompted deeper emotional and cognitive engagement, particularly when participants reflected on their networks. These patterns suggest that tablet-based methods might be more suitable for large-scale studies where efficiency is key. There is a further paradoxical consideration: For some studies on sensitive topics or with vulnerable populations (e.g., those involving bullying or abuse, or where social desirability bias is likely), greater emotional engagement may itself not be desirable (Ryan and Agoston 2013). Digital methods that generate emotional distance might, in fact, enhance the experience.

In contrast, paper-based methods may be more suitable for in-depth data collection where deeper cognitive and emotional participant engagement are sought. This suggestion comes with the proviso that researchers should be aware that increased engagement may sometimes lead to negative emotional reactions from participants, especially as they reflect on their final networks. More generally, there is an ethical imperative to consider this last stage of any data collection process—the emotional impact on respondents of reflecting on the information they have provided—which may be especially substantial in the context of social networks (Ryan and Agoston 2013). Such impact may not, in fact, be surprising for both participants and researchers, but it can also fundamentally challenge participants’ effort of self-presentation (D’Angelo and Ryan 2021). Further conceptual and empirical work is needed on how best to provide support in such situations.

In addition to our substantive findings regarding network data collection modalities, our study demonstrates the analytical merits of a journey-based approach to conceptualizing data collection processes that can provide nuanced information about respondents’ experiences. It can also be a useful analytical tool for comparing different data collection methods, especially by going beyond measuring data quality to consider participants’ interview experiences. Further exploration of journeys as a tool for analyzing data collection methods seems warranted.

## Strengths and Limitations

Our study has the strength of being respondent-focused and using within-subject randomization to avoid learning effect biases. However, it also has some limitations. The use of focus group discussion may have led to convergence in reporting; future studies could add individual follow-up interviews to capture more diverse views. Our focus on young people in this setting, most of whom had substantial past smartphone experience, may limit generalizability to older or less digitally literate populations. It is also plausible that participants’ experiences may be a function of the specific wording using during the interview process and the local context. Therefore, it might merit further comparative work using different interview protocols or different contexts. The work presented here is largely explorative and qualitative, thus future work could benefit from a more statistically powered approach to enable statistical inferences as well. Finally, the networks elicited were moderate in size, with an average of around 12 alters; the trade-offs in method may look different for smaller core networks or larger extended ones.

## Conclusion

In this study of personal social network data collection methodologies, we argue that researchers should move beyond a narrow understanding of participants’ experiences as mere technology users and highlight our congruent ethical duty to address both the positive and negative emotional impacts of network data collection on participants. We show that digital methods might be more suitable for high-volume social personal network projects or for sensitive topics, where less emotional engagement might be desirable. In contrast, paper methods might fit better within qualitative and in-depth studies given their deeper emotional and cognitive engagement. Finally, we showcase the merits of a journey-based analytical approach to better understand data collection methods from respondents’ point of view.

## Appendix

### Standardized Map drawings

- Please name people who are important in your life for any reason, starting with the most important. Write in black pen.
- Is there anyone else who you can confide in or talk to about your problems? Put in red.
- Is there anyone else who could help you with tasks around the home? Put in blue.
- Suppose you need to borrow some small thing like a tool or a cup of sugar, from who outside your household would you ask to borrow it? Put in purple.
- Is there anyone else who you enjoy spending time with socially? Put in pink.
- Is there anyone else with whom you communicate rarely, but when you do it makes a big difference in your life or theirs? Put in green.
- Is there anyone else who is especially close to you but you have not listed previously? Put in orange.

### Friend Map

Please place each person on this map, placing those who are more important to you closer to the center. You can also put people who are closer to each other closer together.

- Please highlight anyone who lives in the same homestead as you. Circle in cerise pink
- What is this person’s age? In red
- Is this person male or female? Fe for Female, M for Male in Black pen

About how often do you see this person?

ED Every day or almost every day

FTW A few times a week

OW Once a week

FTM A few times a month

OM Once a month

LM Less than once a month

About how often do these two people talk to one-another (either in person or otherwise)?

Every day or almost every day ___________________

A few times a week - - - - - - - - - - - - - - - - -

Once a week 

A few times a month 

Once a month . . . . . . . . . . . . . . . .

Less than once a month 

Is this person a member of your family or not?

Family member F in navy

Not a family member NF in navy

Does this person live in the same *isigodi* as you?

Same isigodi circle in bottle green or yellow

Elsewhere (work—navy)/ (school—red)

Several of the workshops described in this study.
